# Prevalence and clinical factors that are associated with low health-related quality of life in children with vascular malformations: a cross-sectional study

**DOI:** 10.1097/MS9.0000000000005421

**Published:** 2026-08-06

**Authors:** Stefanos Intzes, Christos Gkogkas, Trey L. DeJong, Stavros Tombris

**Affiliations:** a Hemangioma and Vascular Malformations Clinic, Iaso Pediatric Hospital, Marousi-Athens, Greece; b Mitera Hospital, Pediatric and Adolescent Oncology Clinic, Marousi-Athens, Greece; c Washington State University School of Medicine, Spokane, WA, USA; d Department of Mathematics and Statistics, Washington State University, Pullman, WA, USA; e Department of Maxillofacial Surgery Iaso Pediatric Hospital, Marousi-Athens, Greece

**Keywords:** children, cross-sectional, Greece, health-related quality of life, OVAMA, vascular malformations

## Abstract

**Background:**

Patient-reported health-related quality of life (HR QoL) outcomes are important for determining the burden of disease and responses to therapy in patients with vascular malformations (VaMs).

**Methods:**

A cross-sectional observational study of 28 children with VaM was performed to evaluate the prevalence of and factors associated with low HR QoL by administering the disease-specific Outcome Measures for Vascular Anomalies and PROMIS measures to assess psychosocial and physical domains.

**Results:**

We detected impairments in various measures of HR QoL, particularly pain interference and difficulty with peer relationships, and noted that the type of VM (particularly AVM) and larger lesion size (>10 cm) were associated with worse pain, appearance, and overall general symptoms; depressive symptoms and anxiety were not prevalent in young children but were prevalent in adolescents.

**Conclusions:**

The small sample size affects the reliability of the subgroup analyses, but the data add to the limited pediatric literature on HR QoL in patients with VaM by emphasizing the importance of early screening for pain symptoms and the emergence of affective disorders, thereby promoting prompt interventions.

## Introduction

Vascular malformations (VaMs) arise due to dysregulation of blood vessel growth during embryonic development and include low-flow lesions [such as venous malformations (VMs), lymphatic malformations (LMs), and capillary malformations], high-flow lesions [arteriovenous malformations (AVMs)], and combinations thereof. A variety of overgrowth syndromes often involve VaMs. Patients with VaM report increased pain and impaired mental health, as noted in patient-reported health-related quality of life (HR QoL) outcome measures^[^1–6^]^. Pediatric data are limited, with inconsistent findings regarding the prevalence of impaired HR QoL^[^3,7^]^. We strove to address the knowledge gap in children regarding pain, mental health, and other important HR QoL measures by using novel, specifically derived, validated questionnaires for this patient population. Previous studies have mostly applied nonspecific tools to the VaM population. The validated Outcome Measures for Vascular Anomalies (OVAMA)^[^8^]^ questionnaire was developed to address a core outcome measurement set, including patient-reported domains [General Symptoms, Appearance, and Patient-Reported Outcomes Measurement Information System (PROMIS^®^) scales] and clinician-reported signs. The goal of the study was to investigate the prevalence of poor HR QoL in children with VaM and to explore associated clinical factors. Since this was the first time we employed the OVAMA questionnaire, we wanted to evaluate the feasibility of its use in the clinic.

HIGHLIGHTS

- Patient-reported health-related quality of life (QoL) outcomes are an integral part of care delivery, particularly in patients suffering from chronic illnesses, such as vascular malformations (VaMs). Still, there is a paucity of data regarding the prevalence and factors that affect QoL in pediatric patients within this population. We performed a cross-sectional observational study of children with VaM by administering the disease-specific Outcome Measures for Vascular Anomalies (translated into Greek) and PROMIS tools to assess psychosocial and physical domains. We detected impairments in various measures, particularly pain interference and difficulty with peer relationships, even at a young age. Our results add to the literature on the QoL of children with VaM and emphasize the importance of early screening for pain symptoms and the emergence of affective disorders.

## Materials and methods

A cross-sectional observational pilot study was performed, involving 28 patients aged 5–17.999 years with VaM, recruited over a 6-month period. The study received Institutional Review Board approval, and informed consent was obtained. The OVAMA questionnaire^[^8^]^ was translated into Greek following the creators’ instructions. The PROMIS^®^ Pediatric Profile GenPop v3.0 Profile-48, assessing Physical Mobility, Anxiety, Depressive Symptoms, Fatigue, Peer Relationships, and Pain Interference, was also administered to patients aged ≥8 years. The Parent-Proxy equivalent was used for patients 5–7.999 years. The OVAMA mean General Symptoms and mean Appearance scores were converted to composite values, with scores >50 considered elevated. PROMIS^®^ T scores were considered elevated if >60 (1 SD above the mean). Higher values indicated greater levels of the measured construct. Potentially contributory clinical factors (Table 1) were extracted from the medical records and de-identified. Variable selection was based on factors that were identified in other analyses, such as pain, location of the VaM, therapy used^[^2,7^]^, and elements that were deemed to be of interest, including sex, type of malformation, presence of overgrowth, muscle involvement, lesion size, decreased function, hospitalizations unrelated to therapy, presence of localized intravascular coagulation, and urban versus rural setting as a social determinant. The type of VaM was classified according to the 2024 International Society for the Study of Vascular Anomalies classification. Given the rarity of VaM and the 6-month recruitment period in this pilot study, we could not attain the desired sample size. Using G*Power (version 3.1.9.7)^[^9^]^ statistical software, and assuming that the current sample of 28 was split into two equal comparison groups for a parametric *t*-test, with α = 0.05 and power = 0.8, a minimum Cohen’s *d* effect size of 1.10 would be necessary for adequate power. Statistical analyses, including *t*-tests, one-way ANOVA, and their nonparametric counterparts, were performed via JASP. Effect sizes and confidence intervals are reported given the limited statistical power of the current sample and serve as a reference point for future power analyses. In cases with statistically significant *P*-values but confidence intervals suggesting otherwise, the confidence intervals use a strict Bonferroni correction, whereas the *P*-values do not. Tukey post hoc corrections were applied to all follow-up comparisons in the one-way ANOVA.

**Table 1:** Demographic information and clinical characteristics of the study VaM patients.

Factors	Number (%)
**Sex**	
Female	20 (71)
Male	8 (29)
**Median age (years)**	11
**Age 5–7.999**	9 (32)
**Age 8–11.999**	6 (21)
**Age 12–17.999**	13 (47)
**Age range (years)**	5–17.999
**Location**	
Head and neck	11 (39)
Torso	4 (14)
Upper extremities	4 (14)
Lower extremities	6 (21)
More than one location	3 (12)
**Type of VaM**	
VM	16 (57)
LM	4 (14)
AVM	4 (14)
Mixed	3(11)
GVM	1(4)
**Size**	
>10 cm	10 (36)
5–10 cm	12 (43)
<5 cm	6 (21)
**Affected type of tissue**	
Skin/subcutaneous	28 (100)
Muscle	16 (57)
Bone	1(4)
Viscera	1(4)
**Overgrowth**	3(11)
**Underlying syndrome**	3(11)
**Chronic pain**	16 (57)
**Decreased function**	13 (46)
**Bleeding**	1(4)
**Urban residence**	17 (61)
**Therapy in past 12 months**	20 (71)
**Targeted therapy**	4 (14)
**Interventional radiology**	17 (61)
**Surgery**	11 (61)
**LIC**	4 (14)

AVM, arteriovenous malformation; GVM, glomuvenous malformation; LIC, localized intravascular coagulation; LM, lymphatic malformation; VaM, vascular malformation; VM, venous malformation.

## Results

We investigated 28 patients (Table 1). The prevalence of poor HR QoL was elevated in psychosocial domains, with impairments in anxiety (11%), fatigue (11%), depressive symptoms (18%), peer relationships (43%), pain interference (46%), general symptoms (32%), and appearance (50%).

**Figure 1.**
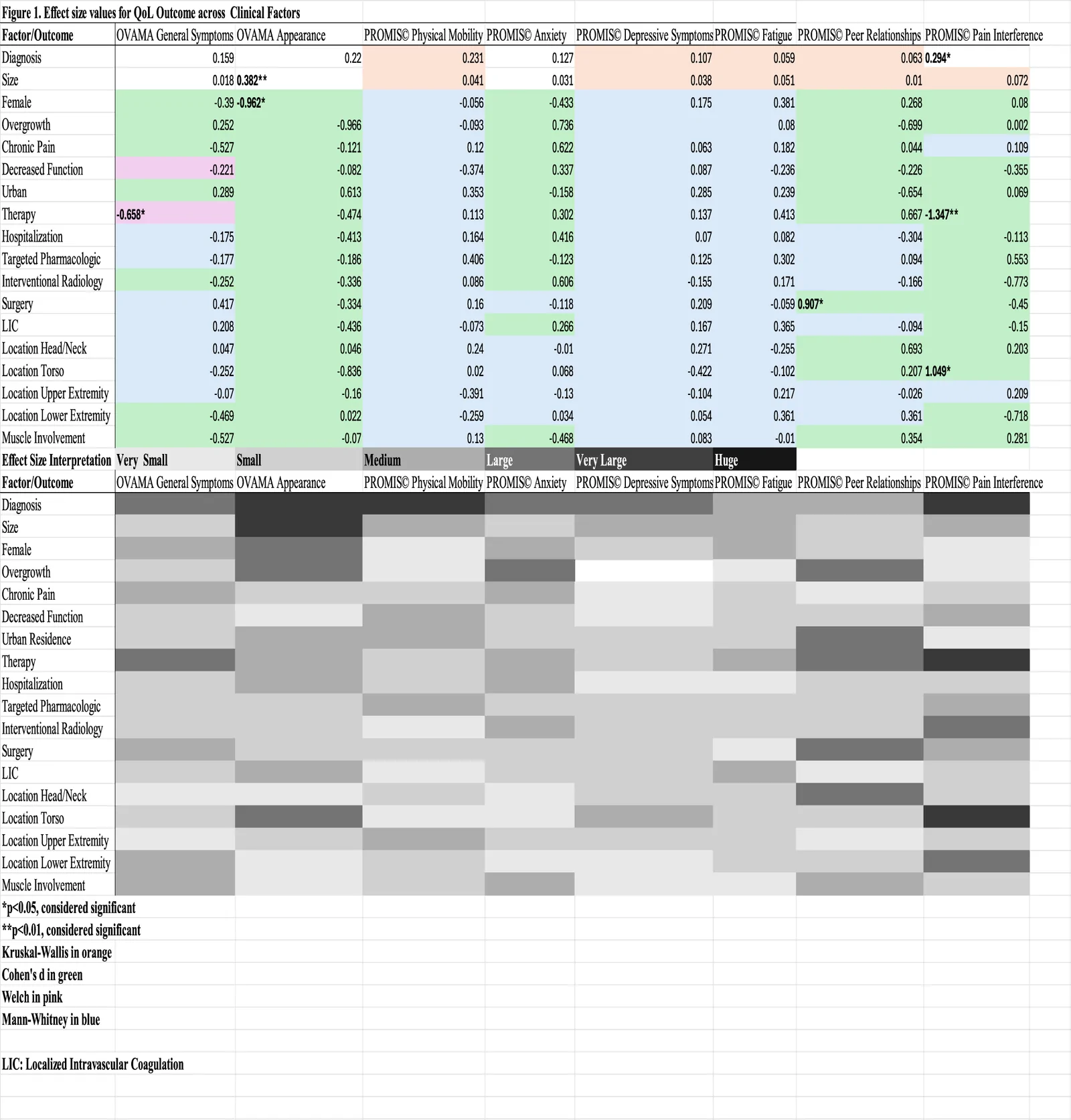
Effect-size values for the QoL outcome across clinical factors.

Figure 1 presents the effect sizes and significance of clinical factor comparisons. Effect size guidelines^[^10^]^ display results based on conventional effect size interpretation, revealing the magnitude of the finding regardless of the number of patients. We detected a statistically significant relationship between the type of diagnosis and pain interference [*P* = 0.042, η^2^ = 0.294 (95% CI: 0.000–0.519)] as well as general symptoms [*P* = 0.049, η^2^ = 0.285 (95% CI: 0.000–0.412)], size >10 cm and impaired appearance [*P* = 0.002, η^2^ = 0.382 (95% CI: 0.076–0.594)], female sex and impaired appearance [*P* = 0.030, *d* = −0.962 (95% CI: −1.814, −0.094); (having received recent], therapy and general symptoms [*P* < 0.044, Hedges’ *g* = −0.776 (95% CI: −1.619, 0.084)] as well as pain interference [*P* = 0.003, *d* = −1.347 (95% CI: = −2.233, −0.439)], location (torso) and pain interference [*P* = 0.024, *d* = 1.049 (95% CI: 0.138–1.941)], and surgery with peer relationships [*P* = 0.027, *d* = 0.907 (95% CI: 0.102–1.696)]. For the Tukey’s honestly significant difference post hoc test for diagnosis and pain interference, AVM showed substantially and statistically significantly higher pain interference than LMs [*P* = 0.039, *d* = −2.041 (95% CI: −4.259, 0.177)]. Including the above findings as well as some non-significant observed effects, many notable effect sizes were found. Torso and lower extremity locations [*P* = 0.112, *d* = −0.718 (95% CI: −1.589, 0.165)], therapy, IR [*P* = 0.056, *d* = −0.773 (95%CI: −1.553, 0.020)], and diagnosis type showed large effects on pain interference. Urban residence [*P* = 0.103, *d* = −0.654 (95% CI: −1.427, 0.130)], therapy [*P* = 0.123, *d* = 0.667 (95% CI: −0.178, 1.501)], surgery, head and neck (H&N) location [*P* = 0.081, *d* = 0.693 (95% CI: −0.085, 1.458)], and overgrowth [*P* = 0.263, *d* = −0.699 (95% CI: −1.905, 0.520)] demonstrated large effects on peer relationships. Size, type of diagnosis [*P* = 0.120, η^2^ = 0.220 (95% CI: 0.000–0.448)], and torso location [*P* = 0.067, *d* = −0.836 (95% CI: −1.713, 0.056)] manifested large appearance effects, while type of diagnosis and therapy had a large effect on general symptoms.

## Discussion

This is one of the first studies to implement OVAMA and PROMIS^®^ questionnaires to explore HR QoL in children with AVM. Stor *et al*^[^11^]^ employed the same tools and included 47 children to investigate appearance concerns and the impact on HR QoL. They reported that body distortion and being stared at correlated with impaired peer relationships. Appearance issues increased with age, and dissatisfaction in adulthood led to anxiety and depression. We observed impaired HR QoL in psychosocial domains, particularly in patients with AVM. AVM is highly destructive to neighboring tissues and leads to pain, disfigurement, and bleeding. These results will need to be confirmed in a larger sample but underscore the importance of monitoring and managing pain at all ages. Physical function mobility was not impaired in our population, in contrast to adult data^[^11^]^, reflecting the resilience of youth and the natural history of the condition. It was interesting that an H&N lesion was not associated with a worse appearance score. However, peer relationships are of particular importance in children, and they were influenced by involvement of the H&N. Although fatigue, depression, and anxiety were not reported in high numbers, all the patients demonstrating such symptoms were older, as seen in adults^[^11^]^.

While the reported effect sizes may assist future researchers with power analyses when studying these outcomes, the estimates may lack precision because of the small samples on which they are based. However, the reported confidence intervals should provide context for the precision of these estimates, using the current data to understand the plausible range of values these effects might take in the target population. Most study patients had recently received therapy, which may have introduced referral bias.

## Conclusion

The small sample size does not permit definitive conclusions, particularly for subpopulation analyses. Nonetheless, these data suggest impaired HR QoL in children with VaM, and more research is required to confirm these findings. We recommend increased vigilance and early implementation of measures to address pain, anxiety, depression, and detrimental effects on peer relationships, even in young children. The questionnaires are feasible to administer in a multidisciplinary clinical setting and will be essential for prospective evaluation of therapy effects.

## Ethical approval

Ethical approval was obtained via Institutional Review Board evaluation.

## Data Availability

Data are available upon request. All authors contributed to the collection and analysis of the data.

## Research registration unique identifying number (UIN)

Research registration was not applicable.
